# The Effectiveness of Traditional Chinese Medicine in Treating Patients with Leukemia

**DOI:** 10.1155/2016/8394850

**Published:** 2016-10-25

**Authors:** Yu-Jun Wang, Chung-Chih Liao, Hsuan-Ju Chen, Ching-Liang Hsieh, Tsai-Chung Li

**Affiliations:** ^1^Graduate Institute of Integrated Medicine, China Medical University, Taichung, Taiwan; ^2^Graduate Institute of Chinese Medical Science, China Medical University, Taichung, Taiwan; ^3^Management Office for Health Data, China Medical University Hospital, Taichung, Taiwan; ^4^College of Medicine, China Medical University, Taichung, Taiwan; ^5^Department of Chinese Medicine, China Medical University Hospital, Taichung, Taiwan; ^6^Research Center for Chinese Medicine & Acupuncture, China Medical University, Taichung, Taiwan; ^7^Department of Healthcare Administration, College of Health Science, Asia University, Taichung, Taiwan; ^8^Department of Public Health, College of Public Health, China Medical University, Taichung, Taiwan

## Abstract

Leukemia is the most common malignancy among all childhood cancers and is associated with a low survival rate in adult patients. Since 1995, the National Health Insurance (NHI) program in Taiwan has been offering insurance coverage for Traditional Chinese Medicine (TCM), along with conventional Western medicine (WM). This study analyzes the status of TCM utilization in Taiwan, in both pediatric and adult patients with leukemia. A retrospective cohort study was conducted using population-based National Health Insurance Research Database of Registry of Catastrophic Illness, involving patient data from 2001 to 2010 and follow-up data through 2011. The effectiveness of TCM use was evaluated. Relevant sociodemographic data showed that both pediatric and adult patients who were TCM users one year prior to leukemia diagnosis were more likely to utilize TCM services for cancer therapy. A greater part of medical expenditure of TCM users was lower than that of TCM nonusers, except little discrepancy in drug fee of adult patients. The survival rate is also higher in TCM users. Altogether, these data show that TCM has the potential to serve as an adjuvant therapy when combined with conventional WM in the treatment of patients with leukemia.

## 1. Introduction

Leukemia originates in the bone marrow and results in the production of a large number of abnormal white blood cells. The four main subtypes of leukemia are acute lymphoblastic leukemia (ALL), acute myelogenous leukemia (AML), chronic lymphocytic leukemia (CLL), and chronic myelogenous leukemia (CML) [1]. Leukemia is the most common malignancy diagnosed in children accounting for greater than 30% of all childhood cancers [2]. While ALL is the most prevalent cancer in childhood and also the first leading cause of death from cancer within 20 years of age [3], AML is more common in older adults [4]. Patients with leukemia may undergo chemotherapy or hematopoietic stem cell transplants, which are associated with massive physical suffering and economic burden [5, 6].

The National Health Insurance (NHI) program has offered insurance cover for Traditional Chinese Medicine (TCM) and conventional Western medicine (WM) since 1995. Several studies have demonstrated that TCM as adjuvant therapy can help improve the quality of life and alleviate the side effects associated with WM use. Importantly, it can enhance the efficacy of WM treatment against tumors and improve the survival rate [7–13]. For instance, one study indicated that TCM combined with chemotherapy significantly elevates survival rate and improves the quality of life in patients with advanced non-small-cell lung carcinoma [12]. Another observational study demonstrated that TCM as adjuvant therapy helps to lower the risk of mortality relative to “TCM nonusers” in advanced breast cancer [9]. Apart from case reports and standardized questionnaire surveys, there are few large-scale studies on TCM use among leukemia patients [14, 15]. Thus, our study aimed to explore the determinants of TCM utilization in both pediatric and adult leukemia patients and further evaluate the overall survival of patients.

## 2. Materials and Methods

### 2.1. Research Data Sources

The NHI program, initiated in 1995, currently covers nearly 99.6% of the residents in Taiwan. The Bureau of National Health Insurance has contracts and cooperative agreements with nearly 97% of all hospitals and 92% of all clinics. This study included National Health Insurance Research Database (NHIRD) claim datasets that are safe from counterfeits due to the severe penalties involved [16]. The NHIRD datasets used in this study consisted of registry for beneficiaries, ambulatory and inpatient care claims, and registry for catastrophic illness from 1999 to 2011. Besides these, the NHIRD database contains demographic data, dates of visits, International Classification of Diseases, Ninth Revision, Clinical Modification (ICD-9-CM) diagnostic codes, complete prescription details, and expenditure incurred by the beneficiaries. Data for detailed diagnoses and treatments provided by physicians were included. On the other hand, all cancer cases registered in the catastrophic illness database were eligible for an exemption from copayment. Our study was approved by the Joint Institutional Review Board of Public Health, Social and Behavioral Science Committee, China Medical University and Hospital.

### 2.2. Study Population

Our population-based retrospective cohort study included newly diagnosed patients with leukemia (ICD-9-CM code 204–208) identified from the registry of catastrophic illness between January 1, 2001, and December 31, 2010. In Taiwan health care system, the diagnosis of leukemia cases was done by WM physicians and cannot be done by TCM physicians because all diagnosis tests, including regular blood routine test (CBC/DC), blood smear (observing blood cell morphology), bone marrow biopsy (observing bone marrow cell morphology), and symptoms and signs, can be performed only by WM physicians. The index date was the initial date of leukemia diagnosis and all patients were followed up through December 31, 2011, or until the patients' death date within this interval or withdrawal from NHI. The index date was the date of first diagnosis. We analyzed 2,355 newly diagnosed pediatric patients (0–18 years old) and 10,208 newly diagnosed adult patients (19–80 years old) with leukemia. Patients who visited TCM physicians and used TCM at least once after being diagnosed with leukemia were deemed “TCM users” and the rest were deemed “TCM nonusers.”

### 2.3. Comorbidities and Classification of Expenditures

This study considered TCM and WM ambulatory care, including dates of visit, date of birth, patient gender, medical facility and department visited, prescribing physician, dispensing pharmacist, three items from the ICD-9-CM codes, primary procedure, type of copayment, and paid amounts. We also analyzed the coexisting diseases in the leukemia patients based on the ICD-9-CM codes. Types of expenditure included fees for consultation, treatment and medical supplies, diagnosis fee, and drug fee.

### 2.4. Sociodemographic Factors and Urbanization Levels of Residential Area

**Figure 1:**
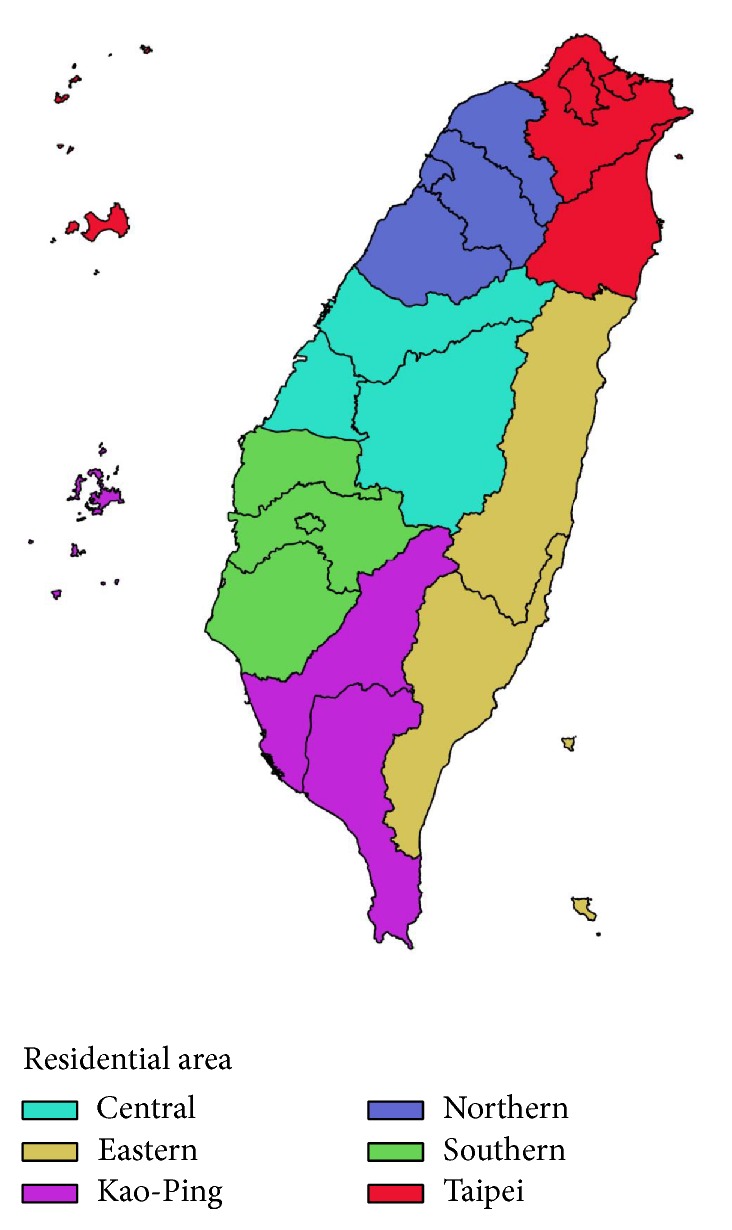
The residential areas of the study population.

The sociodemographic factors included age, gender, insurance premium amount, and the insured unit. Pediatric patients were further divided into 3 subgroups: 0–6, 7–12, and 13–18 years. Similarly, the adult patients were also divided into 3 subgroups: 19–40, 41–60, and 61–80 years. The amount of insurance premium, determined from the individual working salary, was classified into four levels: <20,000, 20,000–39,999, 40,000–59,999, and >60,000 NT$/month. The residential areas of the study population comprised 6 areas: Northern area, Taipei, Central area, Southern area, Eastern area, and Kao-Ping area (Figure 1). Furthermore, the urbanization level of the townships in Taiwan was categorized according to educational level of the population, population density, the ratio of elder people, and occupation in general [17]. The insured unit included government, school, private enterprise, occupational member, farmer and fishermen, low-income household, and veterans.

### 2.5. Statistical Analysis

All analyses were performed separately for the pediatric and adult patient groups. The continuous variables were evaluated using means, standard deviations, and 95% confidence interval (CI), whereas categorical variables were evaluated using the numbers, percentages, and 95% CI. To compare the differences in continuous variables between TCM users and nonusers, Student's *t*-test was used, whereas Chi-square test was used to analyze the categorical variables. Furthermore, the adjusted odds ratio (OR), calculated using a multivariate logistic regression analysis, was used to explore the determinants of TCM utilization. The Kaplan-Meier estimator with a log-rank test was applied to evaluate the effect of TCM use on overall survival. Pairwise comparisons of overall survival among groups of no, low, and high TCM use were adjusted by Bonferroni correction. All *p* values were calculated using two-sided tests, and the threshold for statistical significance was set at *p* < 0.05. All analyses were performed using Statistical Analysis System (SAS) version 9.3 (SAS Institute Inc., Cary, NC, USA).

## 3. Results

### 3.1. Factors Associated with TCM Use

A total of 292 (12.40%) pediatric patients and 936 (9.17%) adult patients availed TCM outpatient services. In these leukemia patients, we observed that patients who were TCM users one year prior to the leukemia diagnosis and who resided in central area were more likely to utilize TCM services (Table 1). The mean numbers of days taking TCM in pediatric and adult leukemia patients were 184 (SD = 328) days and 107 (SD = 254) days, respectively. The mean outpatient department (OPD) visits for TCM in pediatric and adult leukemia patients were 20.3 (SD = 35.3) and 10.5 (SD = 22.8), respectively. The distributions of cancer type were similar between TCM users and nonusers in both pediatric and adult leukemia patients.

### 3.2. Medical Institutes

TCM users within pediatric leukemia patients engaged private hospitals more often for outpatient services than TCM pediatric nonusers (42.15% versus 38.70%). Similar trend was also seen within adult leukemia patients (36.17% versus 34.20%). On the contrary, private clinics were more often engaged for outpatient services by TCM nonusers of pediatric patients when compared to TCM users (41.37% versus 35.22%) as well as by TCM adult nonusers (43.32% versus 41.13%) (Table 2).

### 3.3. Coexisting Diseases

We observed that lymphoid leukemia and myeloid leukemia were the two major diagnoses in these leukemia patients. Lymphoid leukemia was common in children and myeloid leukemia was diagnosed mainly in adults. Acute upper and lower respiratory infections were commonly diagnosed in both pediatric and adult patients, irrespective of TCM use by the patients (Table 3).

### 3.4. Expenditures

According to our analysis, drug fee was the major component of all outpatient medical expenditures (Table 4). For both pediatric and adult leukemia patients, the average cost of total amount per visit was higher for TCM users than that of TCM nonusers. In adult leukemia patients, the average cost of drug fee per visit in TCM users was higher than that of TCM nonusers. Nevertheless, the differences in average costs between these two groups were indeed minimal (2,557.98 NT$ versus 2,535.07 NT$). The costs of other items per visit in TCM users in adult leukemia patients were significantly lower than those of TCM nonusers, including fees for consultation, treatment, medical supply, and diagnosis fee. In pediatric patients, the average cost per visit in TCM users was consistently higher than that of TCM nonusers for all items including fees for consultation, treatment, medical supply, diagnosis fee, and drug fee.

### 3.5. Most Commonly Prescribed TCM Single and Formula Products

Details of the TCM single and formula products most frequently prescribed by TCM physicians are shown in Table 5. In pediatric leukemia patients,* Radix Astragali membranaceus* was the most commonly prescribed TCM single product, followed by* Bulbus Fritillariae thunbergii*, and* Herba Hedyotis diffusa*. Moreover, Xiang Sha Liu Jun Zi Tang was the most frequently prescribed TCM formula product, followed by Yu Ping Feng San and Zuo Gui Wan. The top three most commonly used TCM single products for adult leukemia patients were as follows, from the most common to the least:* Radix Astragali membranaceus, Radix et Rhizoma Salviae miltiorrhizae*, and* Fructus Ligustri Lucidi*. Moreover, the top three most commonly prescribed TCM single products were Gui Pi Tang, Liu Wei Di Huang Wan, and Zuo Gui Wan.

### 3.6. Overall Survival

**Figure 2:**
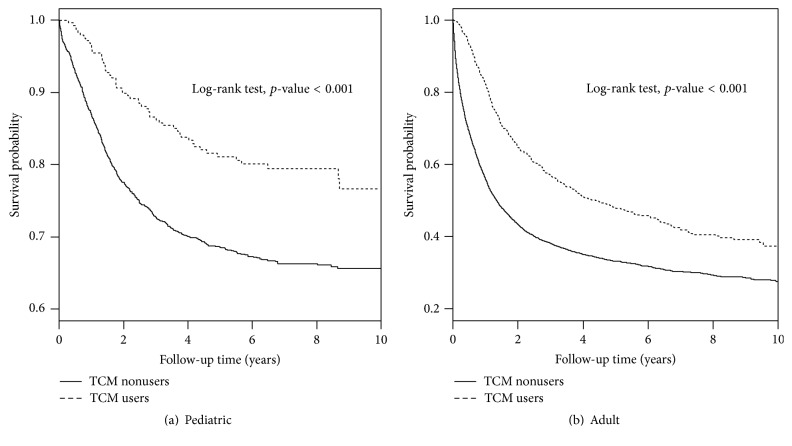
Kaplan-Meier curves of overall survival in patients with leukemia based on TCM use during the follow-up period.

**Figure 3:**
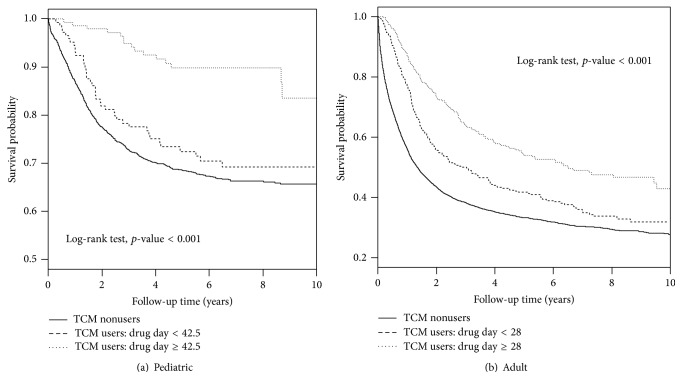
Kaplan-Meier curves of overall survival in patients with leukemia based on number of drug days for TCM use during the follow-up period. (a) TCM nonusers versus TCM user: days taking TCM < 42.5, *p* = 0.0051. TCM nonusers versus TCM user: days taking TCM ≥ 42.5, *p* < 0.001. TCM user: days taking TCM < 42.5 versus TCM user: days taking TCM ≥ 42.5, *p* = 0.0049. (b) TCM nonusers versus TCM user: days taking TCM < 28, *p* < 0.001. TCM nonusers versus TCM user: days taking TCM, *p* < 0.001. TCM user: days taking TCM < 28 versus TCM user: days taking TCM ≥ 28, *p* = 0.0001.

**Figure 4:**
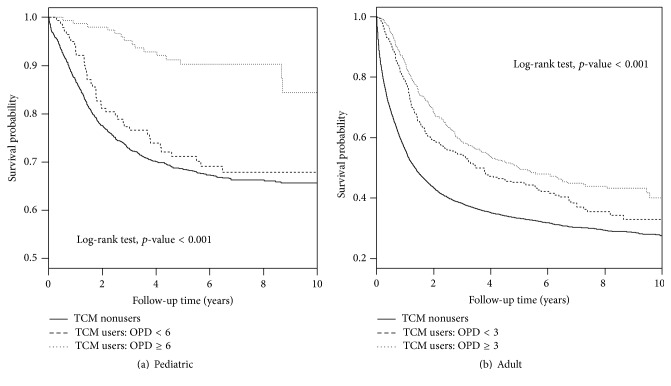
Kaplan-Meier curves of overall survival in patients with leukemia based on number of outpatient department (OPD) visits for TCM use during the follow-up period. (a) TCM nonusers versus TCM user: OPD < 6, *p* = 0.0075; TCM nonusers versus TCM user: OPD ≥ 6, *p* < 0.001; TCM user: OPD < 42.5 versus TCM user: OPD ≥ 6, *p* = 0.0009. (b) TCM nonusers versus TCM user: OPD < 3, *p* < 0.001; TCM nonusers versus TCM user: OPD ≥ 3, *p* < 0.001; TCM user: OPD < 3 versus TCM user: OPD ≥ 3, *p* < 0.001.

We found that the overall survival rate was higher in TCM users compared to TCM nonusers in both pediatric and adult patients with leukemia (both *p* < 0.001) (Figure 2). During the 10 years that we followed, both pediatric and adult patients with leukemia who were TCM users had near 10% higher survival probability in comparison to the corresponding group of TCM nonusers patients. To further examine whether the overall survival rates were influenced by the number of days taking TCM and number of OPD visits for TCM use, we categorized these two variables using their median values as cutoff points (Figures 3 and 4). We observed that the overall survival rates were significantly different in subgroups of number of days taking TCM and OPD visits for TCM use in both pediatric and adult patients with leukemia (all *p* < 0.001). We observed a dose-response relationship among three subgroups of TCM use according to either patients with number of days taking TCM or number of OPD visits for TCM use. The highest overall survival rates were found in patients with high number of days taking TCM (≥42.5 for pediatric patients and ≥28 for adult patients), which were significantly higher than those with low number of days taking TCM (<42.5 for pediatric patients and <28 for adult patients) (*p* = 0.0049 for pediatric patients and *p* < 0.0001 for adult patients) and were also significantly higher than those who were TCM nonusers (both *p* < 0.001 for pediatric and adult patients). The differences in overall survival curves were different between patients with low number of days taking TCM and TCM nonusers (*p* = 0.0051 and <0.001 for pediatric and adult patients, resp.). Similarly, we observed significant pairwise differences in overall survival function among three subgroups of TCM use based on number of OPD visits.

## 4. Discussion

This study investigated the potential of TCM use as an adjuvant therapy for leukemia patients, both pediatric and adult, in Taiwan undergoing treatment with WM. The determinants for TCM utilization for leukemia patients in Taiwan, such as medical expenditure, and therapeutic effects were only scarcely reported in the past [18]. Our study shows that the combination therapy using TCM and WM for patients with leukemia does not cause additional financial burden relative to using WM only. Importantly, TCM use is associated with longer survival.

We observed that TCM utilization was more prevalent in pediatric leukemia patients rather than adults. We assume that children can barely tolerate the extremely uncomfortable symptoms and feelings accompanying chemotherapy, as previously suggested [19]. Studies show that TCM can alleviate adverse effects of WM and improve quality of life in cancer patients [7, 10, 11, 13]. Andersen's health behavior model, which is advocated by many scholars of sociology and public health, claims that the use of health services is determined by predisposing factors, enabling factors, and need factors [20]. In our study, significant factors for TCM use included predisposing factors of gender and health beliefs, such as TCM use one year prior to leukemia diagnosis. Enabling factors observed were height of medical insurance, residential area, and urbanization level, which were similar to those reported by Liao et al. [21, 22]. On the other hand, a particularly higher prevalence of TCM utilization was observed in central Taiwan, which has the highest per capita ratio of TCM physicians.

*Radix Astragali membranaceus* and* Fructus Ligustri Lucidi* were in the top 3 herbs for adult users and* Radix Astragali membranaceus* was the top one herbs for pediatric users. Both of these herbs have a function that increases white blood cell count [23]. Due to the indication of leukemia being a consistently elevated white blood cell count, some physicians thought using the herbs that increase white blood cell count is not recommended because of the potential exacerbation of leukemia. Given the findings of the present study, these herbs may increase healthy WBC without aggravating the leukemia. This can be supported by the prior studies of these two herbs demonstrating that they bolster host immune response for cytotoxic activity by promoting apoptosis of tumor cells and may help the recovery from chemotherapy or radiation treatments [18, 24–27]. The findings of the present study provide important information for TCM physicians and patients with leukemia.

The National Health Insurance program was started on March 1, 1995. In 2011, 22.60 million out of 22.96 million Taiwan residents were enrolled in this program. The comprehensive NHI database is considered an appropriate source to assess certain diseases. In addition, the NHI database provides abundant sample population size and details in studies, eliminating the bias associated with a limited sample size.

Our study has several limitations, though. Firstly, some patients visiting TCM physicians preferred to receive decoction formulations of Chinese medicine, which were self-paid and not covered by the NHI program. In addition, about 10% of the TCM clinics were not contracted to the NHI because of the low reimbursement rates provided by the NHI. Therefore, the prevalence of TCM use and costs of outpatient visits could be underestimated. Secondly, we only explored the efficacy of general TCM utilization, including overall survival, but we did not consider the subtypes of leukemia and TCM prescriptions, which should be investigated in further studies. Last, the database does not contain information on daily activity, dietary habits, and body mass index, which may also be factors for TCM utilization and health care costs. Further studies considering the above information are therefore warranted.

## 5. Conclusion

Our study investigated the TCM utilization and prevalence among pediatric and adult patients with leukemia in Taiwan. It shows that TCM as adjuvant therapy combined with WM may indeed alleviate adverse symptoms, improve quality of life, and prolong overall survival, without incurring excess medical expenditure. This line of study in the future should aim at ruling out the possible confounders such as diet, lifestyle behaviors of exercise, and psychological status. The results of our study could be useful to health-policy decision makers as well as clinical practitioners while considering the integration of TCM with WM.

## Figures and Tables

**Table 1:** Sociodemographic factors of pediatric and adult patients with leukemia according to use of TCM.

Characteristic	Pediatric (*N* = 2,355)					Adult (*N* = 10,208)				
	TCM users		TCM nonusers		Adjusted OR^‡^ (95% CI)	TCM users		TCM nonusers		Adjusted OR^‡^ (95% CI)
	*N*	%	*N*	%		*N*	%	*N*	%	
Number of patients	292		2,063			936		9,272		
Age, years										
0–6	139	47.60	1,016	49.25	1.00					
7–12	89	30.48	480	23.27	1.10 (0.81–1.50)					
13–18	64	21.92	567	27.48	0.64 (0.46–0.89)^*∗∗*^					
19–40						274	29.27	2,199	23.72	1.00
41–60						379	40.38	3,012	32.48	0.95 (0.80–1.13)
61–80						284	30.34	4,061	43.80	0.60 (0.48–0.70)^*∗∗∗*^
Means (SD)	8.03	(5.07)	8.41	(5.65)		51.26	(15.67)	55.39	(17.24)	
Gender										
Female	129	44.18	843	40.86	1.00	428	45.73	3,740	40.34	1.00
Male	163	55.82	1,220	59.14	0.83 (0.64–1.07)	508	54.27	5,532	59.66	0.86 (0.74–0.99)^*∗*^
TCM use one year prior to leukemia diagnosis										
TCM nonusers	180	61.64	1,523	73.82	1.00	437	46.69	6,048	65.23	1.00
TCM users	112	38.36	540	26.18	1.62 (1.23–2.13)^*∗∗∗*^	499	53.31	3,224	34.77	1.97 (1.71–2.26)^*∗∗∗*^
Insured amount (NT$/month)										
<20,000	286	97.95	2,035	98.64	1.00	610	65.17	6,736	72.65	1.00
20,000–39,999	6	2.05	27	1.31	2.02 (0.79–5.15)	197	21.05	1,613	17.40	1.23 (1.02–1.47)^*∗*^
40,000–59,999	0	0.00	1	0.05	—	101	10.79	735	7.93	1.48 (1.15–1.90)^*∗∗*^
≥60,000	—	—	—	—	—	28	2.99	188	2.03	1.65 (1.08–2.55)^*∗*^
Urbanization										
Level 1 (highest)	78	26.71	564	27.34	1.00	278	29.70	2,521	27.19	1.00
Level 2	97	33.22	655	31.75	0.85 (0.59–1.24)	279	29.81	2,698	29.10	0.86 (0.70–1.04)
Level 3	48	16.44	371	17.98	0.52 (0.32–0.82)^*∗∗*^	149	15.92	1,625	17.53	0.67 (0.53–0.85)^*∗∗∗*^
Level 4	45	15.41	296	14.35	0.63 (0.39–1.02)	134	14.32	1,329	14.34	0.74 (0.58–0.96)^*∗*^
Level 5 (lowest)	24	8.22	177	8.58	0.63 (0.34–1.14)	96	10.26	1,098	11.84	0.69 (0.51–0.94)^*∗*^
Residential area										
Northern	32	10.96	304	14.74	1.00	85	9.08	1,245	13.43	1.00
Taipei	75	25.68	750	36.35	0.76 (0.46–1.24)	274	29.27	229	34.83	1.01 (0.77–1.34)
Central	104	35.62	306	14.83	3.33 (2.12–5.23)^*∗∗∗*^	265	28.31	1,623	17.51	2.41 (1.85–3.14)^*∗∗∗*^
Southern	36	12.33	324	15.71	1.17 (0.69–1.99)	163	17.41	1,372	14.80	1.95 (1.47–2.59)^*∗∗∗*^
Eastern	3	1.03	42	2.04	0.73 (0.21–2.58)	27	2.88	261	2.82	1.78 (1.11–2.84)^*∗*^
Kao-Ping	42	14.38	337	16.34	1.16 (0.70–1.92)	122	13.03	1,541	16.62	1.13 (0.84–1.52)
Insured unit										
Government, school employees	39	13.59	176	9.30	1.00	130	13.92	1,056	11.42	1.00
Private enterprise employees	145	50.52	916	48.39	0.70 (0.47–1.05)	404	43.25	3,533	38.21	0.91 (0.73–1.13)
Occupational member	37	12.89	342	18.07	0.47 (0.28–0.77)^*∗∗*^	198	21.20	1,973	21.34	0.87 (0.68–1.12)
Farmers, fishermen	36	12.54	226	11.94	0.69 (0.40–1.18)	141	15.10	1,827	19.76	0.80 (0.60–1.07)
Low-income households and veterans registered in local government agency	30	10.45	233	12.31	0.58 (0.34–0.98)	61	6.53	857	9.27	0.73 (0.52–1.02)
Number of days taking TCM, means (SD)	184	(328)	—	—		107	(254)	—	—	
OPD visits for TCM, means (SD)	20.3	(35.3)	—	—		10.5	(22.8)	—	—	
Cancer subtype										
Lymphoid leukemia	205	70.21	1,377	66.75	1.00	217	23.18	1,896	20.45	1.00
Myeloid leukemia	79	27.05	609	29.52	1.10 (0.83–1.44)	649	69.34	6,670	71.94	0.85 (0.72–1.00)
Uncertain	8	2.74	77	3.73	0.88 (0.42–1.85)	70	7.48	706	7.61	0.87 (0.65–1.15)

ICD-9-CM: lymphoid leukemia, 204.xx; myeloid leukemia, 205.xx.

TCM: Traditional Chinese Medicine; OR: odd ratio; SD: standard deviation; CI: confidence interval; OPD: outpatient department.

^*∗*^ *p* < 0.05, ^*∗∗*^ *p* < 0.01, and ^*∗∗∗*^ *p* < 0.001.

^‡^Adjusted ORs were from the model considering age, gender, visit one year ago, insured amount, urbanization, residential area, and insured unit.

**Table 2:** Distributions of outpatient service providers for pediatric and adult patients with leukemia during 2001–2011.

Outpatient services providers	TCM users		TCM nonusers		*p* value for *χ* ^2^
	Visits	Percentage (95% CI)	Visits	Percentage (95% CI)	
Pediatric					
Type of provider					<0.001
Public hospitals	20,407	22.33 (22.06, 22.6)	80,522	19.47 (19.35, 19.59)	
Public Chinese medicine hospitals	20	0.02 (0.01, 0.03)	92	0.02 (0.02, 0.03)	
Private hospitals	38,522	42.15 (41.83, 42.47)	160,049	38.70 (38.55, 38.85)	
Private Chinese medicine hospitals	86	0.09 (0.07, 0.11)	191	0.05 (0.04, 0.05)	
Public clinics	160	0.18 (0.15, 0.2)	1,497	0.36 (0.34, 0.38)	
Private clinics	32,187	35.22 (34.91, 35.53)	171,108	41.37 (41.22, 41.52)	
Other medicine service providers	9	0.01 (0, 0.02)	103	0.02 (0.02, 0.03)	

Total	91,391		413,562		

Adult					
Type of provider					<0.001
Public hospitals	52,167	20.64 (20.49, 20.80)	354,202	19.77 (19.71, 19.83)	
Public Chinese medicine hospitals	400	0.16 (0.14, 0.17)	905	0.05 (0.05, 0.05)	
Private hospitals	91,396	36.17 (35.98, 36.36)	612,655	34.20 (34.13, 34.27)	
Private Chinese medicine hospitals	614	0.24 (0.22, 0.26)	4,044	0.23 (0.22, 0.23)	
Public clinics	3,782	1.5 (1.45, 1.54)	40,716	2.27 (2.25, 2.29)	
Private clinics	103,932	41.13 (40.94, 41.32)	776,052	43.32 (43.24, 43.39)	
Other medicine service providers	399	0.16 (0.14, 0.17)	3,026	0.17 (0.16, 0.17)	

Total	252,690		1,791,600		

TCM: Traditional Chinese Medicine; CI: confidence interval.

**Table 3:** Top 5 disease codes among pediatric and adult leukemia patients during the years 2001–2011 for all outpatients visits.

Ranking	TCM users			TCM nonusers		
	Disease (Code)	Number	Percentage (95% CI)	Disease (Code)	Number	Percentage (95% CI)
Pediatric						
Total visits	91,391			413,562		
1	Lymphoid leukemia (204)	36,529	39.97 (39.65, 40.29)	Lymphoid leukemia (204)	138,518	33.49 (33.35, 33.64)
2	Myeloid leukemia (205)	7,801	8.54 (8.35, 8.72)	Acute upper respiratory infections (465)	45,756	11.06 (10.97, 11.16)
3	Acute upper respiratory infections (465)	6,873	7.52 (7.35, 7.69)	Myeloid leukemia (205)	34,551	8.35 (8.27, 8.44)
4	Leukemia of unspecified cell type (208)	4,698	5.14 (5, 5.28)	Acute bronchitis and bronchiolitis (466)	15,506	3.75 (3.69, 3.81)
5	Acute bronchitis and bronchiolitis (466)	2,962	3.24 (3.13, 3.36)	Acute sinusitis (461)	12,141	2.94 (2.88, 2.99)

Adult						
Total visits	252,690			1,791,600		
1	Myeloid leukemia (205)	55,031	21.78 (21.62, 21.94)	Myeloid leukemia (205)	264,653	14.77 (14.72, 14.82)
2	Lymphoid leukemia (204)	19,336	7.65 (7.55, 7.76)	Acute upper respiratory infections (465)	97,491	5.44 (5.41, 5.47)
3	Acute upper respiratory infections (465)	11,117	4.4 (4.32, 4.48)	Lymphoid leukemia (204)	76,117	4.25 (4.22, 4.28)
4	Gingival and periodontal diseases (523)	5,680	2.25 (2.19, 2.31)	Diabetes mellitus (250)	56,042	3.13 (3.1, 3.15)
5	Diseases of hard tissues of teeth (521)	5,123	2.03 (1.97, 2.08)	Essential hypertension (401)	53,009	2.96 (2.93, 2.98)

TCM: Traditional Chinese Medicine; CI: confidence interval.

**Table 4:** Expenditures for outpatient services for pediatric and adult leukemia patients (NT$) during the period 2001–2011.

Characteristic	TCM users			TCM nonusers			*t* value
	Total	Percentage (95% CI)	Average cost per visit (95% CI)	Total	Percentage (95% CI)	Average cost per visit (95% CI)	
*Pediatric*							
Outpatient visits	91,391			413,562			
Fees for consultation, treatment, and medical supplies	52,088,100	38.09 (38.08, 38.1)	569.95 (559.62, 580.28)	245,838,958	32.74 (32.73, 32.74)	594.45 (589.71, 599.19)	4.22^*∗∗∗*^
Diagnosis fee	19,341,052	14.14 (14.14, 14.15)	211.63 (210.86, 212.4)	88,180,707	11.74 (11.74, 11.74)	213.22 (212.84, 213.61)	3.64^*∗∗∗*^
Drug fee	65,311,809	47.76 (47.75, 47.77)	714.64 (680.14, 749.15)	416,969,010	55.52 (55.52, 55.53)	1008.25 (986.8, 1029.7)	14.16^*∗∗∗*^
Total amount	136,740,961	100	1496.22 (1459.61, 1532.83)	750,988,675	100	1815.92 (1793.74, 1838.1)	14.64^*∗∗∗*^

*Adult*							
Outpatient visits	252,690			1,791,600			
Fees for consultation, treatment, and medical supplies	170,260,064	19.55 (19.55, 19.56)	673.79 (663.88, 683.70)	1,282,955,508	20.66 (20.66, 20.66)	716.10 (711.68, 720.52)	7.64^*∗∗∗*^
Diagnosis fee	54,059,202	6.21 (6.21, 6.21)	213.93 (213.46, 214.41)	386,270,204	6.22 (6.22, 6.22)	215.60 (215.42, 215.78)	6.46^*∗∗∗*^
Drug fee	646,375,253	74.24 (74.23, 74.24)	2557.98 (2512.05, 2603.91)	4,541,817,387	73.12 (73.12, 73.13)	2535.07 (2516.85, 2553.29)	−0.91
Total amount	870,694,519	100	3445.70 (3398.59, 3492.81)	6,211,043,099	100	3466.77 (3447.99, 3485.55)	0.81

^*∗∗∗*^ *p* < 0.001.

**Table 5:** Top ten formula CHPs and single CHPs prescribed by TCM physicians to treat pediatric and adult patients with leukemia in Taiwan, 2000–2011.

	Single CHPs		Formula CHPs	
	Name	Number, frequency (%)	Name	Number, frequency (%)
Pediatric	*Radix Astragali membranaceus*	103 (5.80)	Xiang Sha Liu Jun Zi Tang	36 (7.86)
	*Bulbus Fritillariae thunbergii *	87 (4.90)	Yu Ping Feng San	27 (5.90)
	*Herba Hedyotis diffusa *	74 (4.16)	Zuo Gui Wan	25 (5.46)
	*Radix Scutellariae baicalensis*	65 (3.66)	Bu Zhong Yi Qi Tang	24 (5.24)
	*Radix Puerariae lobatae*	57 (3.21)	Shen Ling Bai Zhu San	20 (4.37)
	*Radix Glycyrrhizae uralensis*	52 (2.93)	Bao He Wan	18 (3.93)
	*Herba Houttuyniae cordata*	45 (2.53)	Xin Yi San	16 (3.49)
	*Semen Ziziphi Spinosi *	34 (1.91)	Ma Zi Ren Wan	16 (3.49)
	*Rhizoma Polygonati odorati*	32 (1.80)	Liu Wei Di Huang Wan	14 (3.06)
	*Herba Scutellariae barbatae*	29 (1.63)	Ge Gen Tang	14 (3.06)

Adult	*Radix Astragali membranaceus*	173 (4.07)	Gui Pi Tang	83 (6.18)
	*Radix et Rhizoma Salviae miltiorrhizae*	133 (3.13)	Liu Wei Di Huang Wan	53 (3.95)
	*Fructus Ligustri Lucidi *	108 (2.54)	Zuo Gui Wan	39 (2.91)
	*Sargentodoxa Cuneata*	86 (2.02)	Gui Lu Er Xian Jiao	39 (2.91)
	*Herba Hedyotis diffusa*	82 (1.93)	Sheng Mai San	36 (2.68)
	*Rhizoma Dioscoreae polystachya*	71 (1.67)	Jia Wei Xiao Yao San	35 (2.61)
	*Bulbus Fritillaria thunbergii*	69 (1.62)	Xue Fu Zhu Yu Tang	27 (2.01)
	*Cortex Lycii Radicis *	63 (1.48)	Xiang Sha Liu Jun Zi Tang	26 (1.94)
	*Radix Glycyrrhizae uralensis*	59 (1.39)	Xin Yi Qing Fei Tang	25 (1.86)
	*Semen Ziziphi Spinosae *	58 (1.36)	Ren Shen Yang Rong Tang	24 (1.79)

TCM: Traditional Chinese Medicine; CHPs: Chinese herb products.
